# Dive into Single, Seek Out Multiple: Probing Cancer Metastases via Single-Cell Sequencing and Imaging Techniques

**DOI:** 10.3390/cancers13051067

**Published:** 2021-03-03

**Authors:** Shang Su, Xiaohong Li

**Affiliations:** Department of Cancer Biology, College of Medicine and Life Sciences, The University of Toledo, Toledo, OH 43614, USA; shang.su@utoledo.edu

**Keywords:** cancer metastasis, single-cell sequencing, single-cell imaging

## Abstract

**Simple Summary:**

Treating cancer metastasis is the biggest challenge in clinical practice. It is largely due to our limited understanding of the complex process, which involves not only the evolution and/or selection of heterogeneous primary tumor cells to metastatic tumors, but also interaction and/or adaption with various types of cells in different microenvironments, including temporally in the circulation system. These limitations are currently resolved by single-cell technologies. This review summarizes recent applications of single-cell technologies in metastatic studies, highlights the unique findings, and discusses the future directions.

**Abstract:**

Metastasis is the cause of most cancer deaths and continues to be the biggest challenge in clinical practice and laboratory investigation. The challenge is largely due to the intrinsic heterogeneity of primary and metastatic tumor populations and the complex interactions among cancer cells and cells in the tumor microenvironment. Therefore, it is important to determine the genotype and phenotype of individual cells so that the metastasis-driving events can be precisely identified, understood, and targeted in future therapies. Single-cell sequencing techniques have allowed the direct comparison of the genomic and transcriptomic changes among different stages of metastatic samples. Single-cell imaging approaches have enabled the live visualization of the heterogeneous behaviors of malignant and non-malignant cells in the tumor microenvironment. By applying these technologies, we are achieving a spatiotemporal precision understanding of cancer metastases and clinical therapeutic translations.

## 1. Introduction

Metastasis is the leading cause of cancer death and has long been a major issue in cancer research [1,2]. Although metastasis is usually found at later or advanced stages, it might occur prior to or at the same time as primary tumor diagnosis [3]. Cancer metastasis involves a series of events known as the metastatic cascade. Briefly, cancer cells detach from the primary tumor mass and enter the circulation, i.e., intravasate primarily into the bloodstream but may also enter the lymphatic system. These cancer cells are named circulating tumor cells (CTCs). The CTCs extravasate, i.e., exit from the vessels when they arrive at distant sites such as the bone, brain, lung, or liver. These cells are then named disseminated tumor cells (DTCs). DTCs adhere and colonize at the distant organs. They may keep proliferating to detectable micrometastases and macrometastases, or stay dormant for years and even decades until being activated/reactivated for proliferation [1,4,5]. The phylogeny of metastasized tumors has been proposed through genetic studies; we also know that many factors, including tumor-intrinsic factors and those from the host microenvironment, control and regulate the metastasis [4]. Bulk metastatic tumor studies helped us sketch the scenarios of metastasis and identify common molecular biomarkers and therapeutic targets. However, genetic heterogeneity is diluted. On the one hand, druggable genomic and transcriptomic alterations are diverse and may represent only small subsets of patients in certain tumor types, which limits their clinical readout in biomarker-driven clinical trials [5]. On the other hand, we could have missed some cells with low proportions which could escape therapy and grow into resistant or relapsed tumors [6]. Moreover, macrometastases may have already undergone some essential but transient events along the metastasis cascade from colonization to micrometastases. Due to these unknown essentials, there is no precision therapy to specifically prevent or target metastasis. Therefore, advanced technologies and approaches are needed to break the limitations of our understanding of the biology of metastasis and to develop novel and effective therapeutic strategies to prevent and cure metastasis. 

The applications of single-cell techniques allow one to decipher how heterogeneous cancer cells interact with various non-malignant cells in the tumor microenvironments under different stresses such as irradiation or treatments. Single-cell analyses can be carried out on both the cell behaviors and the intracellular changes, including the genome transcriptome, proteome, and metabolome. This review will focus on the recent advances in applications of single-cell sequencing and imaging, separately or in combination, in studies of cancer metastases.

## 2. Single-Cell Sequencing

### 2.1. The Overall Experiment Flow

The high accuracy and specificity of next-generation sequencing (NGS) facilitate the high-throughput characterization of RNA expression and DNA alteration. Single-cell sequencing (SCS) is a collective term for sequencing methods on DNA and RNA at the single-cell resolution. Such methods have been developed to amplify the input with the lowest biases while maintaining the high-throughput coverage of genomic and transcriptomic information. Currently, SCS can be performed to examine the status of DNA, RNA, and proteins [7]. Various SCS platforms share a common workflow, as outlined below.

#### 2.1.1. Single-Cell Capture and Nucleic Acid Isolation

**Figure 1:**
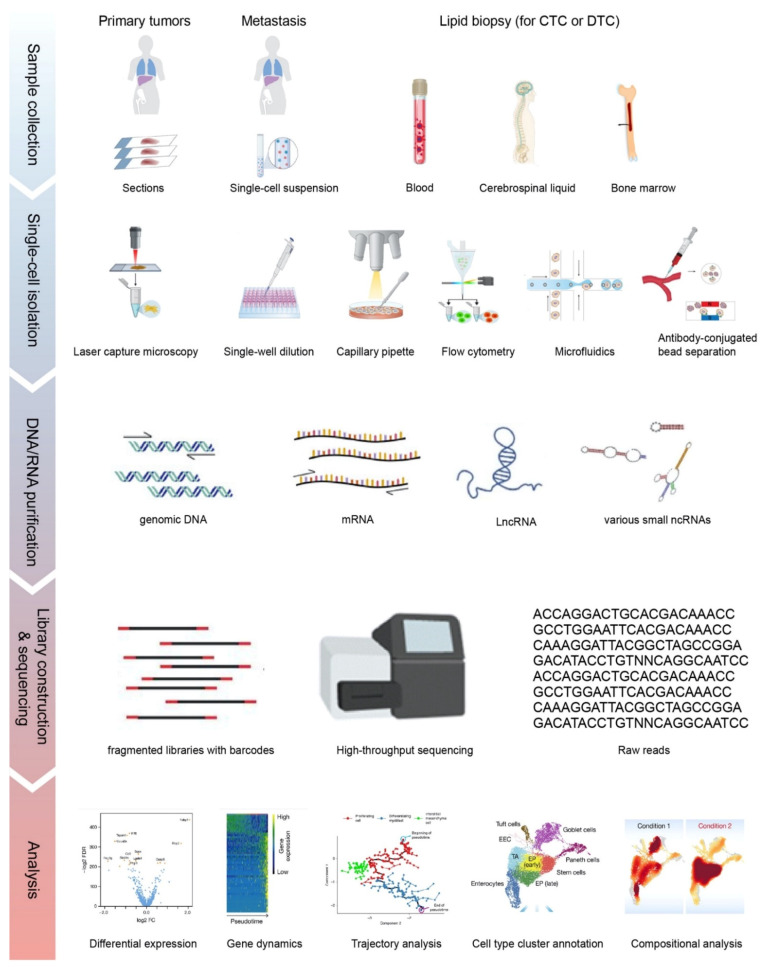
The overall workflow of single-cell sequencing (SCS). Single cells are isolated from solid tissues or liquid biopsies via different approaches. Libraries generated from purified nucleic acids are sequenced on the 2nd or 3rd generation sequencing platforms. Raw reads are processed and visualized as shown in different applications in the Analysis section, including marker gene selection by differential expression, pseudotime tracking by trajectory analysis and cell type cluster to elucidate the population. Parts of the subplots are adapted from the figures in the reference literature [11,12,13]. CTC: circulating tumor cell; DTC: disseminated tumor cell.

The first step for SCS is single cell isolation, which can be carried out through micromanipulation (capillary pipette), laser capture, flow cytometry sorting, or microfluidic devices, depending on the type of starting materials (Figure 1). Recent technical advances also allow CTC or DTC isolation via liquid biopsy including peripheral blood, bone marrow, and cerebrospinal fluid [8,9,10]. The direct isolation of a single cell nucleus is preferred when unprocessed mRNA or genomic information is to be collected. Nonetheless, the isolation methods dictate the final output in terms of purity and detection capacity. 

For the isolation of CTCs, the epithelial cell adhesion molecule (EpCAM) is usually adopted as a marker to capture CTCs and to minimize blood cell contamination. However, it is only applicable for CTCs with a high expression of EpCAM. To capture CTCs with a low expression of EpCAM, microfluidic devices and size-based selection approaches are applied [14]. With size-based selection, more CTCs and their heterogeneities are captured, but contamination from other types of cells is not excluded.

#### 2.1.2. Library Preparation and Sequencing

The library preparation protocols vary on the downstream sequencing platforms. DNA or cDNA from each cell can be labeled with barcodes and then pooled together for sequencing. Most of the SCS methods are based on next-generation sequencing with typical read lengths of 50–150 bp, while SCS approaches such as SMART-Seq and SMART-Seq2 that are powered by third-generation sequencing (also known as long-read sequencing) can process longer reads of up to several kb. The extracted nucleic acids from single cells have to be fragmented to a certain required size range by physical sheering or chemical ion-based methods prior to the adaptor ligation. In addition, unique molecular identifiers of 4–10 bp are introduced to each transcript during reverse transcription to distinguish the reads between amplified copies of the same mRNA molecule and other mRNA molecules transcribed from the same gene. 

#### 2.1.3. Data Processing 

The workflow for SCS data processing can be simplified into three steps:

- File conversion. Convert the raw reads from any SCS machinery to a standardized format, such as fastq.
- Demultiplexing, dataset quality control (QC), filtering, and alignment. Reads can be aligned to a reference genome or transcriptome via typical aligners such as STAR or HISAT2, or by pseudo-aligners such as Kallisto [15,16,17].
- Data visualization and interpretation. Multiple open-source software packages are available for the visualization of cell clusters and subpopulation identification, i.e., Seurat, t-SNE, and UMAP [18,19,20].

The analysis pipeline used for SCS experiments is variable and can be customized based on the research objectives. Those interested in getting into this growing field can refer to two systematic reviews of bioinformatic tools for SCS [11,21]. To be noted, although RNA sequencing is mostly used to assess gene expression, it can also give information about genetic variants. Multiple packages have been developed, including inferCNV, CONICSmat and CaSpER, to identify copy number variations (CNVs) from single-cell RNA-Seq (scRNA-Seq) data to infer mutational phylogenies of cancer cell populations across different samples [22,23,24].

### 2.2. Single-Cell RNA-Seq (scRNA-Seq) in Metastatic Studies

The first SCS study was reported in 2009, in which a single-cell whole transcriptome sequencing protocol was developed to analyze transcriptome complexity in a single mouse blastomere. 75% more gene expressions were detected compared to using the microarray [25]. Since then, scRNA-seq has been advanced to easier cell isolation, higher cell throughput, greater gene coverage, longer read length, and less bias. The advances of scRNA-Seq also benefit metastatic studies.

#### 2.2.1. Spatial Understanding: Mapping the Diversities of the Metastatic Microenvironment

ScRNA-seq allows for high-resolution analyses of the cellular constitution of metastatic tumors in the tumor microenvironment, including fibroblasts, endothelia, various immune cells, etc. As a result, the complexities and dynamics of the metastasis ecosystem can be explored. Studies using this approach have been conducted on various metastases of many cancer types, for example, transcriptome profiling the transcriptomes of nearly 6000 single cells of 18 head and neck squamous cell carcinoma patients, including five matched pairs of primary tumors and lymph node metastases. The stromal and immune cells shared expression patterns across patients, but malignant cells varied within and between tumors. Cells expressing the partial epithelial-to-mesenchymal transition program spatially localized to the leading edge of primary tumors and this program served as an independent predictor of nodal metastasis, grade, and adverse pathologic features [26]. A single-cell transcriptome study in liver metastatic colorectal cancer tissues (2770 cells) and adjacent normal liver tissues (2391 cells) from one patient revealed granulocyte enrichment in the liver metastases and discovered a positive correlation between ferroptosis-mediated cell death and hyperactivated Wnt signaling in the enriched granulocytes [27]. A study comparing the cellular composition and transcriptional states in matched samples of metastatic prostate cancer cells and adjacent bone marrow in the spinal cord, as well as bone marrow from orthopedic patients without malignancy, identified that the metastatic tumors had significant exhaustion of cytotoxic T lymphocytes but an increase in inflammatory lymphocytes and macrophages [28]. This study also found that the increased chemokine CCL20, produced by myeloid cells and its cognate CCR6 receptor on T-cells was associated with repressed immune responses, suggesting this might be the cause of the poor response to immune therapy by metastatic prostate cancer, as reported in recent clinical trials [29,30]. A study on myeloma found that CXCL12, a key molecule involved in CXCR4-dependent cell retention in bone marrow, was upregulated in circulating plasma cells and potentially induced myeloma cells’ intravasation [31]. 

Besides mapping the diversity of tumor microenvironment, scRNA-Seq is also helping to identify new therapeutic targets for metastasis. A recent study on uveal melanoma, which is highly metastatic, interrogated the tumor microenvironment (TME) at a single-cell resolution using scRNA-seq of ~60k tumors and non-neoplastic cells from primary and metastatic samples [32]. They found that among the tumor-infiltrating immune cells, the CD8+ T cells had only minimal expressions of CTLA-4 and PD-1 but the strongest expression of LAG3. This indicates T cell exhaustion and may partially explain the failure of targeting CTLA-4 and/or PD-1 in uveal melanoma. LAG3 blockade could be a potential effective immune therapy for these patients [32].

#### 2.2.2. Temporal Understanding: Identifying Drivers of Metastasis

Beyond the established metastatic tumors, circulating tumor cells (CTCs) serve as the bridges and messengers between primary tumors and metastatic tumors. Unlike the transcriptomic gene expression profiling of bulk CTC samples, scRNA-seq is able to exclude the contamination from nonmalignant cells and cover the full-spectrum of CTC heterogeneity. The first scRNA-seq in CTC was conducted using SMART-Seq to characterize full-length mRNA profiles from melanoma patient samples and identified distinct potential metastatic biomarkers in CTC such as CDH1 and HLA1 [33]. By comparing the genome-wide expression profiles of CTCs with matched primary tumors in a mouse model of pancreatic cancer, another study discovered that CTCs clustered separately from primary tumors and tumor-derived cell lines, and they presented a low-proliferation signature [34]. The aberrant expression of stromal extracellular matrix genes by CTCs revealed the relevance of epithelial to mesenchymal transition (EMT) and contributions of microenvironmental signals to metastasis [34]. Novel findings were made in CTCs. For example, the elevation of noncanonical Wnt signaling (Wnt5a) was found to be associated with anti-androgen resistance through scRNA-seq of 77 CTCs from 13 prostate cancer patients [35]. Markers of cancer stem cells (CSCs) and of EMT were found in breast cancer through scRNA-seq of 666 CTCs from 21 breast cancer patient samples [36]. A consistent induction of β-globin (HBB) was observed in CTCs across breast, prostate, and lung cancers [37]. HBB was induced by KLF4 upon intracellular reactive oxygen species (ROS), and contributed to the survival of tumor cells under ROS stress, suggesting a cytoprotective effect of the signaling to suppress intracellular ROS during the circulation in the bloodstream [37].

These CTC profiling studies are snapshots of the metastatic cascade. A technical breakthrough, however, is the longitudinal CTC profiling. Through an optofluidic system that continuously collects fluorescently labeled CTCs from a genetically engineered mouse model, the researchers were able to use scRNA-Seq in profiling CTCs isolated longitudinally from the mice over four-day treatments with JQ1, an inhibitor of the bromodomain and extraterminal (BET) family of bromodomain proteins [38]. This is probably the first time the dynamic drug responses in terms of CTCs have been revealed. The future of translating this technology into clinical practice is very exciting. Furthermore, a recent study characterized single-cell profiles of CTCs in the cerebrospinal fluid in lung adenocarcinoma leptomeningeal metastases [9]. This advance provides the opportunity to compare CTCs from blood and CTCs from more local metastatic environments and, thus, the understanding of the site preference mechanisms in metastasis. 

Altogether, these studies demonstrated the power of scRNA-seq in profiling CTCs, which are believed to be one of the best candidates in diagnosis and prognosis in metastasis prevention and treatment. The major challenges are capturing pure CTCs with high quality and translating new technologies into clinic.

### 2.3. Single-Cell Whole Genomic Sequencing and Whole-Exome Sequencing in Metastatic Studies

The next-generation sequencing of bulk tumors suggested that metastasis is initiated by a subclone of the primary tumor, based on their shared genomic alterations between primary and respective metastatic tumors. However, metastatic tumors often have unique mutations or genomic alterations that are not found in the primary tumors. The question is whether the metastasis-exclusive mutations were present below the detection limit in the primary tumor or whether they evolve after leaving the primary site. The single-cell whole-genome sequencing (scWGS) in isolated single nuclei of breast cancer cells was developed in order to answer these questions [39]. This was also the first SCS study in metastasis. Flow-sorted single-nucleus sequencing was performed for a previously identified genetically homogeneous breast duct carcinoma (52 nuclei) and its paired liver metastasis (48 nuclei). It was found that a single clonal expansion from the primary tumor evolved to metastasis. Following this study, single-cell whole-exome sequencing (scWES) was developed in 2012, focusing on protein-coding genomic regions in the metastasis study [40,41].

WGS and WES can profile the genomic landscapes in CTCs and metastatic tumors, including single-nucleotide variants (SNVs), insertions/deletions (indels), copy number alterations/variations (CNAs/CNVs), and the loss of heterozygosity (LOH). Similar to the transcriptomic landscape of CTCs, the intrapatient and interpatient heterogeneity of CTCs at the genomic level are frequently observed in prostate, lung, and breast cancer [42,43,44,45,46]. Using scWGS, androgen receptor (AR) gene positive or negative prostate cancer CTC subpopulations were identified during the period of androgen deprivation therapy (ADT); CNV evolution reflecting clinical response and disease progression was also observed in CTCs [47]. In metastatic breast cancer patients, the majority of CTC mutations that were detected at baseline disappeared; but some mutations were enriched and new mutations emerged during standard treatment, suggesting the evolution or shifting of the CTC population [48]. Other recent studies showed that genomic variations of CTCs represent their competencies of intravasation and migration/motility, abilities of cell–cell interactions, variations of energy metabolism, emergences of blood immune cells, and resistances to anoikis or certain therapy [16,41,42]. 

Furthermore, genomic alterations including CNV can also be inferred from RNA-sequecing data. While scRNA-Seq can provide useful information for characterizing the CNV architecture of essential oncogenes and tumor-suppressor genes, copy numbers of intergenic regions are not well represented. This limitation is also shared by scWES [49]. However, scWGS provides more coverage of genomic information [49]. Therefore, scWGS is recommended to get a much broader spectrum of CNV landscape in metastatic cascade. Recent studies that integrated genomic sequencing data and scRNA-Seq in CNV calling have achieved better information of evolution [22,50].

### 2.4. Single-Cell Epigenomic Sequencing and Emerging Multi-Omics

With the word "epigenome," we refer to all genomic changes that do not alter the primary DNA sequence but nonetheless could be hereditary. Such changes include DNA methylation, histone modification, chromatin binding of structural and regulatory proteins, and chromosome conformation (3D genome). The first methylome profiling for single cells was achieved using single-cell reduced representation bisulfite sequencing (scRRBS) in 2013 [51]. The first single-cell chromosome conformation capture was conducted using high-throughput sequencing (Hi-C) [52]. The first single-cell chromatin immunoprecipitation sequencing (ChIP-seq) was conducted using a combination of microfluidics, DNA barcoding, and next-generation sequencing [53]. These approaches acquired low-coverage maps of H3 lysine-4 tri-methylation (H3K4me3) and di-methylation (H3K4me2) in mixed populations of mouse embryonic stem cells, embryonic fibroblasts, and hematopoietic progenitors. 

The simultaneous profiling of genome, transcriptome, epigenome, proteome, metabolome, or other emerging whole-genome scale information is known as multi-omics. At single-cell level, the approaches (listed in Table 1) include scG&T-seq (single-cell genome and transcriptome sequencing) and scMT-seq (single-cell methylome and transcriptome sequencing). Combinations of various antibodies and massively parallel mRNA sequencing have also been developed recently to simultaneously monitor the transcriptome and a limited panel of surface proteins at single-cell level, including CITE-Seq, REAP-Seq and Ab-Seq (Table 1). Several studies have adopted single-cell epigenomics or multi-omics logics to characterize CTCs or metastatic samples in a limited scale of throughput (only methylation of several genes or certain metabolites, or limited proteins) [54,55,56]. For example, quantitative methylation analysis of nine genes in metastatic breast cancer CTCs isolated from 37 patients showed that patients with methylated CST6, ITIH5, and RASSF1 in CTCs had a significantly shorter progression-free survival relative to patients without these methylations [54]. In a lipid profiling study of CTCs from colorectal cancer and gastric cancer, colorectal cancer CTCs had higher amounts of sterol lipids and acylcarnitine, while gastric cancer CTCs had higher amounts of fatty acids and glycerophospholipids [55]. Simultaneous detections of glucose uptake, key phosphoprotein levels, and EGFR/KRAS mutations were performed on some rare CTCs from the blood sample of a female metastatic lung adenocarcinoma patient [56]. These studies showed the feasibility of using SCS to determine the whole landscape of tumor evolution in metastasis and identify novel vulnerabilities for metastasis prevention and treatment.

### 2.5. Summary and Future Perspectives

Single-cell sequencing has provided unprecedented advantages and resolution in understanding cancer metastasis, including how individual cells of the metastatic tumor evolve, how heterogeneity forms, and how the cells differ in their physiological behavior or responses to therapies, spatially and temporally. Although SCS is not being used as a tool to make treatment decisions just yet, it is paving the way for precision medicine and individualized treatment at a higher level with apparently improved detections at protein, RNA and gene levels. The clinical effectiveness will further validate and improve the models we constructed and interpreted from the single-cell data. While single-cell isolation and low-level DNA/RNA sequencing are always the challenges due to the small inputs, which may generate biases during processing, the technology in the SCS field is actively evolving. The ever-increasing single cell datasets also significantly boost the deconvolution of previously published bulk sequencing data. This enables more answers to the questions that used to be elusive, thus facilitating further and deeper interpretation of the data and translation from basic biology to the clinic. For example,

(1) Are the subpopulations identified in metastatic cancer samples indeed the driving subsets of metastasis or only the endpoint adaptation to their destination microenvironment? This is a long-standing and challenging question. Longitudinal comparisons among samples from distinct metastatic sites of the same primary cancer type or of the same patient, along with the CTCs, may help reconstruct the dynamics of metastasis and elucidate this question.
(2) How are the cells organized spatially in metastatic samples? Spatial transcriptomic methods (such as seqFISH and Slide-Seq) have been developed in recent years [72,73]. Developing these technologies at the single-cell level will allow for the spatial deciphering of metastatic tumors.
(3) How do we explore multi-omics from a single cell? Applying multi-omics technologies in one single cell to co-register information at multiple levels would strongly broaden our understanding of metastasis and the toolbox to fight against it.

## 3. Single-Cell Imaging

### 3.1. Seeing Is Believing: Single-Cell Imaging Basics

Imaging approaches allow one to visualize metastasis in situ/in vivo and answer the questions that are beyond the capability of SCS. For example, what and how do different subtypes of cancer cells distribute spatially in a tumor mass? How do cancer cells connect with adjacent cells in the microenvironment? What types of immune cells were excluded from or infiltrate into the tumor, and when? In clinical practice, multiple imaging tools have been applied in the diagnosis of cancer metastasis, such as positron emission tomography (PET), magnetic resonance imaging (MRI), computed tomography (CT), and the like. However, these imaging modalities are not at a single-cell resolution. The single-cell imaging of cancer cells has been achieved in monolayer cultures with simple microscopy. Current techniques in super-resolution microscopy can even distinguish single-molecule dynamics at a nanometer resolution [74,75]. However, the acquisition of single-cell resolution images in in vivo samples, live or fixed, is far more challenging. Tissue status (live or fixed), label strategies (fluorescent, bioluminescent or others), signal-collecting instruments (balancing between sensitivity and specificity), and post-acquisition processing (3D deconvolution) are all limiting factors when applying the single-cell imaging technique in studying cancer metastasis. 

### 3.2. Know Your Samples: Keep It Live or Get It Fixed?

For fixed sample sections, including patient samples, multispectral imaging with multiple antibodies will create the partial atlas of single-cell protein expressions. For example, the immune cell spatial distribution was charted using seven-plex immunofluorescence for the liver and lung metastases of colorectal cancer and infiltrated lymphocytes were found in the progressive metastatic clones [76]. Nucleic acid hybridization probes (ISH/FISH) could also be applied in sections to determine the differences between samples at the DNA or RNA levels [77,78]. The direct visualization of the deeper/inner parts of an intact tissue or an organ is challenging, because the light or signals will be scattered and hampered. To tackle this issue, one can use either a transparentized tissue/model or an imaging apparatus that can visualize more in-depth with higher penetrance. 

#### 3.2.1. Tissue Clearance

Post-mortem tissue clearance by removing the pigments to yield better transparency allows for the cellular-level optical imaging of intact tissues [79]. For example, CUBIC (clear, unobstructed brain/body imaging cocktails and computational analysis) applies statistical analysis to create 3D maps of cancer cells throughout the body and organs of a mouse after tissue-clearing and scanning them. Such maps allow one to visualize of cancer metastasis and the determine of temporal metastatic events through different mice at various time points [80]. Similarly, a deep-learning-based pipeline named DeepMACT was developed for the automatic detection and quantification of micrometastases from whole body scanning images and therapeutic antibody targeting [81].

#### 3.2.2. Transparent Animal Model

The zebrafish, which has a transparent body, has been widely used for cancer metastasis studies [82]. The zebrafish provides a feasible and cost-effective model to track tumor metastasis at the single-cell level, which allows for long-term monitoring (up to two weeks) to track the early events of metastasis [83]. Cancer cells can be implanted into the circulation of a zebrafish or the perivitelline cavity of zebrafish larvae, allowing one to track cells under microscopy, especially the intravasation and extravasation steps [84,85,86]. In addition, the multiplex labeling and implanting of various cell types provided spatial information on metastasis, and that cancer-associated fibroblasts (CAFs) were found to hijack the dissemination of cancer cells [87]. However, the real challenge of metastasis studies in zebrafish is the limitation in recapitulating metastasis of patients, for which rodents such as rats and mice are more widely used. 

### 3.3. Choose a Label—How to Track Metastatic Cancer Cells in Live Animals?

Intravital microscopy (IVM) imaging of live animals such as rats, mice and zebrafish allows one to track the real-time dynamics of metastases. IVM can image exposed tissues, or it can be carried out through optical windows or endomicroscopy. Imaging through optical windows allows metastasis observations for a relatively long period in vital organs such as the brain, liver, and lung [88,89,90]. Combining fluorescent protein tags or chemical dyes with upgraded fluorescence microscopy can allow metastatic cells to be directly observed in the organs of interest.

#### 3.3.1. Fluorescent Proteins

Tagging cells with fluorescent proteins became available in the 1990s. Using this approach, IVM imaging quickly evolved into an important tool for metastasis observation in the genetic engineered animal models of cancer [91]. Multicolor imaging further enables the comparison of the metastatic potential of multiple clones from the same tumor population [92,93]. Photoswitchable fluorescent proteins, such as Dendra2, which switches from green to red under either visible blue or UV light, have demonstrated that cancer cells were selected during certain steps of the metastatic cascade at a single-cell resolution [94].

#### 3.3.2. Fluorescent Dyes

Fluorescent dyes and probes have also been applied in in vivo imaging. To circumvent the interference of tissue autofluorescence and the tissue scattering of signals, far- and near-infrared trackers (>700 nm) with better specificity and sensitivity are preferred. For example, indocyanine green (~800 nm) tends to accumulate in tumor cells much more readily than in normal cells, and can be applied in fluorescence-guided surgery [95]. In addition, cancer-specific targeted fluorescent agents have been introduced. A targeted tracer consists of a fluorophore conjugated to a targeting moiety, which specifically binds to a marker of cancer cells; thus, the fluorescence will be observed only in cells with the expression of the marker [96]. For example, a fluorescein isothiocyanate (FITC) dye was conjugated to folate, which binds to the folate receptors. Therefore, ovarian cancer cells with high level folate receptors were specifically labeled in the study [97]. While the targeted tracers are not able to track those cancer cells with extremely low or no expression of the targeted proteins, these fluorescent dyes hold more clinical potential than fluorescent proteins, since the latter need to be exogenously overexpressed, which is not feasible in patients.

#### 3.3.3. Bioluminescent Labeling

Bioluminescent imaging (BLI) has been widely applied in cancer metastasis studies [98]. Engineered luciferase and luciferin substrates have been developed to facilitate deep-tissue imaging with fewer cells [99,100]. For example, Akaluc and AkaLumine have been shown to emit significantly stronger and brighter signals than the conventional luciferase/luciferin pair, thus allowing for the detection of signals from as few as a single breast cancer cell trapped in the mouse lung [101]. Furthermore, in combination with genetic engineering, this technique enables the studies of the activation patterns in learning behaviors and the video-rate bioluminescence recording of neurons in the striatum for up to one year, implying future long-term metastasis monitoring [101]. This stands for a substantial advance for small-animal BLI and the potential of studying cancer progression and metastasis, such as CTCs and DTCs.

### 3.4. Signal Capture: The Choice of Microscopy in Single-Cell IVM Imaging

Confocal microscopy and multiphoton microscopy have been largely used in IVM imaging for tumors that are labeled with fluorescent proteins or stained with specific dyes. Confocal microscopy collects signals from excitation by single-photon absorbance in the focal plane and collects signals only in the focal plane, thus permitting thin optical sectioning, by either a pinhole in the laser scanning mode or a rotating disk with slits or holes in the spinning disk mode. With the use of confocal laser scanning microscopy, single cell metastases were observed in the lung from 3 to 10 weeks post injection of the RCN-9 colon cancer cells labeled with green fluorescence protein (GFP) into the liver parenchyma of male rats [102], as well as in the skull bone marrow on days 0, 3, 7, and 10 post intracardiac injections of the bone-metastatic derivative prostate cancer cell, PC-3-GFP-BM6, into transgenic nude mice with red fluorescence protein (RFP) [103]. However, the cons of this approach are: (1) fluorescence is generated via the excitation of light throughout the sample, and thus, the specimen can be bleached or damaged; and (2) a compromise of tissue penetration is the result of photons spreading from deep within the specimen experience scattering and rejection. 

In contrast to single-photon confocal microscopy, multiphoton microscopy applies photons with longer wavelengths (and, thus, with lower energy); fluorophores are only excited by absorbing the energy of two or more photons simultaneously. Only the area proximal to the focal plane with a high photon density can be excited, so no more pinholes are needed to exclude non-focal signals. Additionally, the longer wavelength lights in multiphoton microscopy penetrate deeper, typically up to 2 mm below the tissue surface, and scatter less, thus there is less phototoxicity and photobleaching, allowing imaging for a longer time [91]. The responses of liver metastatic tumor cells and host stromal cells to chemotherapeutics in living mice were observed at the single-cell level after the red fluorescent protein-expressing human colorectal cancer cells (HT29) were inoculated into the spleen of green fluorescent protein-expressing nude mice [104]. GFP- or RFP-labeled T-cell lymphoma infiltration into the colon blood vessels of black C57BJ/6 mice was also observed using similar approaches [105]. Furthermore, the long-wavelength lights can produce second-harmonic generation (SHG) to image collagen fibers and allow the imaging of extracellular matrix changes (such as collagen stiffness) which is one of the hallmarks in cancer metastasis [106]. The amount and condition of the extracellular matrix adjacent to carcinoma cells can be directly observed and relatively quantified via the SHG results [107]. 

While multiphoton microscopy provides single-cell images with more tissue depth and less phototoxicity, the resolution at the focal plane is slightly lower than that of confocal microscopy, and pigmented samples may suffer from physical cellular damage through heating. Selective/single plane illumination microscopy (SPIM, also known as light sheet fluorescence microscopy, LSFM) is an alternative optical-sectioning approach used for imaging thick samples [108]. Light sheet microscopy uses a thin sheet (plane) of light for sample excitation (in contrast to a single-photon in confocal) and a second, separate light path for image detection to sidestep image blur created by traditional epi-illumination techniques. The result is an optically sectioned image without the need for a confocal pinhole. In addition, the sheet of light used for sample excitation dramatically reduces sample photo damage. 

### 3.5. Specific Applications in the Near Future

Single-cell imaging is challenging at any depth of a specimen, and there is no single perfect approach for every situation. Various single-cell imaging approaches introduced above collectively enable the direct observation of metastatic cells intravasating, extravasating, and seeding into secondary sites. Multidimensional atlases of metastasis cancers include x, y, z, time course and different markers by multiplex labeling. Given the inspiring achievement of single-cell imaging techniques, future directions for single-cell imaging in metastatic studies are suggested below:

#### 3.5.1. Noninvasive Single-Cell-Resolution Clinical Tools

Clinical tools such as CT, PET, and MRI are capable of providing noninvasive images, but they lack the resolution necessary to visualize the earliest seeding events, because a single pixel may encompass hundreds or thousands of cells. However, these tools hold the capacity to detect signals from a single cell. A brain-seeking clone of MDA-MB-231BR human breast cancer cells was magnetically labeled with fluorescent magnetic particles and injected into the left ventricles of mice, and MRI signals were able to be acquired multiple times from day 0 to day 33 post injection and provided the longitudinal tracking of individual cell fates [109]. A “cellular GPS” via PET/CT was reported in tracking a single breast cancer cell with radioisotope nanoparticle incorporation from tail vein injection to lung arrest [110]. Single-cell tracking using these methods could be applied to determine the kinetics of cell trafficking and arrest in the metastatic cascade, although it remains to be validated whether the signals from the later metastatic foci are too strong to distinguish the signals from single cells. Nonetheless, once these methods can be readily applied in patients as a single-cell resolution, we will get direct dynamics of cancer metastasis.

#### 3.5.2. Simultaneous Monitoring of Cellular Status during Imaging 

Within a tumor population, different cells likely display various states of metabolism and proliferation, which determine tumor progression and therapeutic responses. The time-lapse monitoring of the behaviors of cancer cells engineered with fluorescence ubiquitination cell cycle indicator (FUCCI) in mice via IVM, allow us to determine the cellular status of cancer cells during the interactions with infiltrating blood vessels and the progression to chemotherapy resistance [111]. More bio- and chemical-sensors of biological processes beyond the cell cycle, need to be developed and implemented into metastatic imaging.

#### 3.5.3. Drug Bio-Distribution and -Action in Tumors

The evaluation of the drug–target engagement is essential in characterizing the response and administrating to those best responsive patients. Recently, an accumulation of HER2-targeting mAb trastuzumab (Herceptin) in tumor-associated phagocytes was observed when the AlexFluor647-conjugated mAb was injected into female mice bearing HER2-GFP breast cancer subcutaneous xenografts. Intravital multiphoton microscopy was used in this study to monitor tumor uptake of the mAb [112]. Compared to the conventional isotope labeling and mass spectrometry approach in pharmacodynamics, imaging tools offer more convenience and more layers of information in inter- and intratumor heterogeneity. More feasible imaging tools to evaluate the drug efficacy and distribution in terms of metastatic cancers need to be developed or adopted in the near future.

#### 3.5.4. Emerging Imaging Techniques

Several emerging multiplex imaging techniques have also been applied in cancer studies. Distinct from typical multispectral immunofluorescence with limited spectrum, multiplex imaging techniques acquire information on more proteins on the same tissue section, frozen or fixed. These approaches include CyCIF (CyClic ImmunoFluorescence), CODEX (CO-Detection by indEXing), IBEX (Iterative Bleaching Extends Multiplexity) and MIBI (Multiplexed ion beam imaging) [113,114,115]. CyCIF and IBEX use similar logic of consecutive staining and quenching to repeatedly acquire images with antibody staining on the same section, resulting in imaging of up to 65 proteins; CODEX uses antibodies tagged with unique DNA oligonucleotides for subsequent staining, rather than direct labelling of fluorophores or rare metal elements, to acquire information on 40 targets; MIBI use metal-isotope-labeled antibodies in combination with time-of-flight mass spectrometry to simultaneously track up to 100 targets and can reach a sub-cellular resolution [116,117,118,119,120]. Although these techniques have not been widely applied in metastatic tumor samples yet, their potentials are highly expected. 

#### 3.5.5. Integrated Approaches of Imaging and Sequencing

An early example is SCOPE-Seq, which combined single-cell imaging and barcoded single-cell sequencing in a microwell assay, although it was not able to provide spatial information or the physiological state of cell phenotype [121]. A recent study integrated droplet scRNA-Seq, spatial transcriptomics, and MIBI to compare primary cutaneous squamous cell carcinoma and matched normal skin [122]. The authors identified three keratinocyte populations that were similar to the normal skin and a tumor-specific keratinocyte population that resided within a fibrovascular niche at leading edges of the tumors [122]. These specialized keratinocytes interacted as a hub with basal and adjacent stromal and immune cell types to exhibit invasive and immunosuppressive features, with the enrichment of integrin signaling genes ITGB1, FERMT1, and CD151 [122]. These integrated approaches and beyond are expected to be applied in metastatic studies in the near future.

## 4. Summary and Perspective

Here, we reviewed the major applications of single-cell techniques in cancer metastatic studies. SCS and imaging techniques have been significantly advanced in the recent 20 years. These approaches provide us with multiscale, multi-omic information on the metastatic cascade and allow for advances in basic knowledge and clinical translation for the detection, tracking, and treatment of metastasis. Multidimensional atlases of metastasis are being achieved through co-applications of single-cell techniques with multiple approaches such as epigenomes and metabolomes. The success of these technologies in solving biomedical questions needs more outstanding co-operations of experts from the various disciplines, including physics and mechanics for the devices, chemists for the labeling and probing, bioinformatics and biostatistics for data analyses, as well as biologists for data interpretation. Furthermore, the recent incorporation of machine learning and AI are offering new directions and are believed to advance cancer research in its translational directions further.

## Figures and Tables

**Table 1:** Summary of major types of SCS approaches used in current research applications.

Research Applications	Representative Approaches	References
Genomic DNA profiling (SNV, CNV, CNA, etc.)	Typical SCS; single-cell exome sequencing	[39,40,41]
Transcriptome	Various scRNA-seq; Smart-Seq2	[25,57]
Newly synthesized and pre-existing RNAs	NASC-Seq	[58]
Non-coding RNA profiling	SMALL-seq; Holo-Seq	[59,60]
DNA methylome profiling	scBS-seq; scPBAT-seq	[61,62]
Histone modification	scChIP-seq	[53]
Chromatin structure (accessibility and interaction)	scATAC-seq; scHi-C; scDNase-seq	[48,59,60]
Genome and transcriptome	DR-Seq; G&T-Seq	[63,64]
Methylome and transcriptome	scM&T-Seq; scMT-Seq;	[65,66]
Genome, transcriptome and methylome	scTrio-Seq	[67]
Transcriptome and limited number of proteins	CITE-Seq; REAP-Seq; Ab-Seq	[68,69,70]
Transcriptome and chromatin accessibility	Sci-CAR	[71]
